# Prevalent cases in observational studies of cancer survival: do they bias hazard ratio estimates?

**DOI:** 10.1038/sj.bjc.6605062

**Published:** 2009-04-28

**Authors:** E M Azzato, D Greenberg, M Shah, F Blows, K E Driver, N E Caporaso, P D P Pharoah

**Affiliations:** 1 Department of Oncology, Strangeways Research Laboratory, University of Cambridge, Worts Causeway, Cambridge CB1 8RN, UK; 2 Genetic Epidemiology Branch, Division of Cancer Epidemiology and Genetics, National Cancer Institute, National Institutes of Health, Bethesda, MD 20852, USA; 3 Eastern Cancer Registration and Information Centre, Unit C, Magog Court, Shelford Bottom, Cambridge CB22 3AD, UK

**Keywords:** survival analysis, prevalent cases, left truncation, breast cancer

## Abstract

Observational epidemiological studies often include prevalent cases recruited at various times past diagnosis. This left truncation can be dealt with in non-parametric (Kaplan–Meier) and semi-parametric (Cox) time-to-event analyses, theoretically generating an unbiased hazard ratio (HR) when the proportional hazards (PH) assumption holds. However, concern remains that inclusion of prevalent cases in survival analysis results inevitably in HR bias. We used data on three well-established breast cancer prognosticators – clinical stage, histopathological grade and oestrogen receptor (ER) status – from the SEARCH study, a population-based study including 4470 invasive breast cancer cases (incident and prevalent), to evaluate empirically the effectiveness of allowing for left truncation in limiting HR bias. We found that HRs of prognostic factors changed over time and used extended Cox models incorporating time-dependent covariates. When comparing Cox models restricted to subjects ascertained within six months of diagnosis (incident cases) to models based on the full data set allowing for left truncation, we found no difference in parameter estimates (*P*=0.90, 0.32 and 0.95, for stage, grade and ER status respectively). Our results show that use of prevalent cases in an observational epidemiological study of breast cancer does not bias the HR in a left truncation Cox survival analysis, provided the PH assumption holds true.

Observational epidemiological studies of cancer are commonly used to study factors that influence disease risk, with an increasing trend to extend them to study molecular factors that influence prognosis. However, many population-based studies recruit cases at variable times after diagnosis (left truncation). Unlike censored data, where partial survival information is known for all individuals, left-truncated data only include information on prevalent cases that are alive at the time of ascertainment. The absence of this subset of prevalent cases that fail to survive until the sampling date results in a study group biased towards favorable survival; without the employment of proper methods to adjust for left truncation, this can seriously bias the parameter estimate (Keiding, 2005).

Although the theoretical basis for treatment of left-truncated data is well established (Brookmeyer, 2005), there are few published examples comparing different analytic approaches to left-truncated data (Cnaan and Ryan, 1989). An example of a population-based study that has been used in left-truncated survival time analyses is the Studies of Epidemiology and Risk factors in Cancer Heredity (SEARCH; Goode *et al*, 2002; Udler *et al*, 2007; Azzato *et al*, 2008; Barnett *et al*, 2008; Song *et al*, 2008). We have extended our studies in the SEARCH breast cancer study to compare the results of survival time analyses (1) restricted to cases enrolled close to the time of diagnosis (limited left truncation) and (2) using cases with substantial left truncation for three well-established prognostic factors – clinical stage, histological grade and oestrogen receptor (ER) status.

## Materials and methods

**Figure 1:**
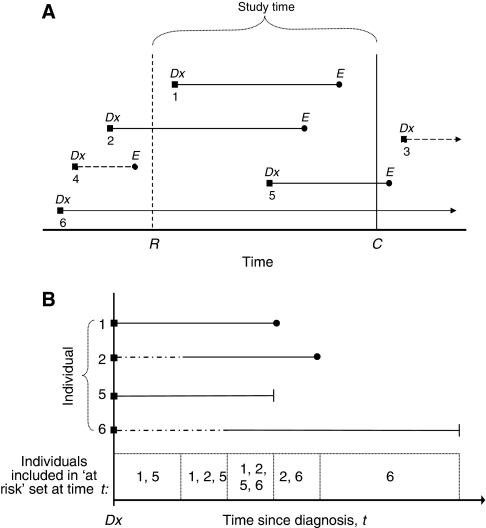
(**A**) Observational epidemiological study with follow-up data, (**B**) survival analysis ‘at risk’ set. (**A**) Study recruitment starts at *R* and ends at *C*. Date of diagnosis and event are indicated by *Dx* and *E* respectively. (**B**) Eligible cases are aligned by *Dx* on *y* axis of time since diagnosis. Dashed lines indicate unobserved time.

Figure 1A provides a schematic of an observational study with follow-up data that include prevalent and incident cases. Study time begins on the date of patient recruitment, *R*, and continues until censoring date, *C*. Participants whose dates of diagnoses (*Dx*) occur within the active study period are incident cases. Participants whose dates of diagnoses occur before patient recruitment are prevalent cases. Time at risk ends at the occurrence of the event of interest, *E*, or at time of censoring, *C*. Some individuals were recruited into the study after their date of diagnosis, *Dx*, whereas other individuals' event dates, *E*, occurred before study recruitment and were not included in the study. The standard Kaplan–Meier estimate of the survival curve will overestimate the true survival curve because it would improperly account for time at risk in left-truncated cases.

Figure 1B shows the individuals and their respective survival times that would have been sampled in our observational study; here we have aligned their survival experiences to begin at time of diagnosis instead of the calendar time of the study. Allowance for left truncation can be made by defining the set of individuals ‘at risk’ (of death, recurrence, etc) at a given time *t* past diagnosis to only include individuals that have not had an event nor been censored before time *t* and who are under active follow-up at time *t* (Brookmeyer, 2005). In this case, time at risk begins at time of study recruitment, *R*, and ends at the either *E* or *C*; the time of delayed entry is not considered ‘at risk’. Therefore, in a survival analysis allowing for left truncation, a component of each recruited individual's time following diagnosis is not considered in the analysis ‘at risk’ set if the individual is not yet recruited for the study (delayed entry time, shown by dashed lines); however, this time is used to align properly individuals on the *y* axis of time since diagnosis. Figure 1B also shows how, in a left truncation survival analysis, the ‘at risk’ set for our example observational study changes with time since diagnosis. Given independent delayed entry (no association between study entry time and truncation time), the conditional hazard ratio (HR) calculated in a left truncation survival analysis should be equivalent to the unconditional HR (Klein and Moeschberger, 2003).

Similarly, left truncation must also be accounted for in a proportional hazards (PH) model that compares the hazard in an exposed population with that in an unexposed population. However, when using Cox models, the PH assumption that the relative hazard remains constant over time must hold. Over a limited follow-up the PH assumption can be reasonably robust, but over a longer follow-up the HR of a prognostic variable may change. Under these circumstances, one option is to treat the prognostic factor of interest as a time-dependent variable in an ‘extended’ Cox model. In such a case, we add a term involving the variable and a function of time (a time-varying covariate), extending the simple Cox model. This analysis can be accommodated in most commonly used statistical packages (SAS, STATA and SPSS). Provided the PH assumption holds, the Cox regression HR estimated from left-truncated data should be an unbiased estimate of the true HR; however, inclusion of a time-dependent covariate will change the practical interpretation of the HRs (Klein and Moeschberger, 2003).

Cases were selected from the SEARCH breast cancer study, an ongoing population study of women diagnosed with breast cancer in the region of England included in the Eastern Cancer Registration and Information Centre (ECRIC, formerly East Anglian Cancer Registry). The study started on 1 July 1996. Eligible participants were women diagnosed with invasive breast cancer aged 55 or younger since 1 January 1991 and alive at the start of the study, and women diagnosed with invasive breast cancer under 70 years of age since the beginning of the study. Owing to boundary changes, some cases diagnosed before 1995 were identified by the North Thames Cancer Registry.

Of those eligible to take part, 67% have returned a comprehensive epidemiological questionnaire and 64% have returned a blood sample for genotyping. Response rates were the same for prevalent and incident cases. All participants in the study provided informed consent, and the study was approved by the Eastern Multicentre Research Ethics Committee. These analyses have been limited to the 4470 individuals that have been included in our ongoing studies of genetic susceptibility to breast cancer.

Patient date of diagnosis, *Dx*, in SEARCH corresponds to the date of diagnosis provided by ECRIC. The date of patient blood draw for genotyping is considered time of study recruitment, *R*, as this is the date the patient entered the cohort. An incident case, therefore, would have either very little or no time from date of diagnosis to date of blood draw. Prevalent cases were defined as cases whose time from date of diagnosis until study entry was >6 months. TNM stage (Sobin and Wittekind, 1997) and histopathological grade were obtained through ECRIC. ER status was determined by performing immunohistochemistry on paraffin-embedded sections of breast tumour using the Novocastra clone 6F11. The Allred system (Harvey *et al*, 1999) was used for scoring; scores >2 were considered positive.

The ECRIC and the North Thames Cancer Registry have active follow-up at years 3 and 5 after diagnosis and then at 5-year intervals. Follow-up information and all-cause mortality are obtained by searching hospital information systems for recent visits. If a patient has not had a recent visit, the patient's general practitioner is contacted to obtain the vital status. Death certificate flagging through the Office of National Statistics also provides the registries with notification of deaths. The lag time with this process is a few weeks for cancer deaths and 2 months to a year for non-cancer deaths.

### Statistical methods

We chose to evaluate stage, grade and ER status, as these variables are well-established breast cancer prognosticators. We compared individual models for stage and grade fitted as both continuous and categorical variables. The fit of the two models (categorical *vs* continuous) was similar for both variables, so we chose to perform all the analyses based on the simplest model (continuous). Univariate Cox regression analysis was performed for each prognosticator separately to determine the effect of each factor on survival using three different scenarios: (a) baseline model using incident cases only (no left truncation, considered unbiased in respect to survival analyses); (b) prevalent cases only allowing for left truncation and (c) all cases allowing for left truncation.

For model (a), individual inclusion in the ‘at risk’ set began on date of diagnosis (*Dx*). For left-truncated analyses, models (b) and (c), individual inclusion in the ‘at risk’ set began at time under observation (*R*, study entry, the date of blood sample receipt). Time under observation in models (b) and (c) will correlate with date of diagnosis for incident cases (Figure 1). Follow-up ended on the date of death from any cause (*E*), or, if death did not occur, on 30 November 2006 (*C*). All analyses were censored at 10 years after diagnosis, as follow-up became less reliable after 10 years. The PH assumption for each prognostic factor was assessed under each model visually using log–log plots, as well as tested analytically using Schoenfeld residuals, based on a test of non-zero slope in a generalised linear regression of the scaled Schoenfeld residuals on a function of time. This is equivalent to testing that the log HR function is constant over time (PH assumption); rejection of the null hypothesis indicates a deviation from the PH assumption (Grambsch and Therneau, 1994).

The parameter of interest from each analysis was the *β*-coefficient (natural log of the HR) and associated 95% confidence interval. Robust variances were calculated (Lin and Wei, 1989). The primary test was a test of heterogeneity (1 degree of freedom) for differences between prognosticator parameter estimates in models (b) and (c) compared to the baseline model (a). This comparison was performed by generation of a *z*-statistic, calculated as the difference in *β*-coefficients divided by the square root of the sum of their squared standard errors. In model (c), a likelihood ratio test (1 degree of freedom) was used to test for interaction between case type (prevalent and incident) and the prognosticator. All analyses were performed in Intercooled Stata, version 9.2 (StataCorp LP, College Station, TX, USA). Statistical tests were two-sided with an α-level of 0.05.

## Results

The survival and prognostic characteristics of the 4470 SEARCH participants included in these analyses are described in Table 1. More than 99% of the cases were Caucasian. There were 1231 (27.5%) cases considered to be incident, providing 8517 years at risk, with a median follow-up time of 7.7 years, and 220 deaths before 10 years of follow-up. There were 3239 (72.5%) prevalent cases, providing 16 532 years at risk, with a median follow-up time of 7.2 years and 490 deaths before 10 years of follow-up. Prevalent cases were more likely than incident cases to be diagnosed at a younger age and present with a higher TNM stage.

### Analyses without correcting for time-dependent effects

The results of the three Cox models for each prognostic factor are presented in Table 2. No differences in the parameter estimates for the different models were seen for stage and grade. However, the parameter estimate for the baseline model for ER status was significantly different than those for the other two models (*P*=0.0006 and 0.03 respectively). A test for interaction of case status (prevalent and incident) and prognostic factor in model (c) was significant for ER status (*P*=0.001), but not for stage or grade (*P*=0.14 and 0.07 respectively).

**Figure 2:**
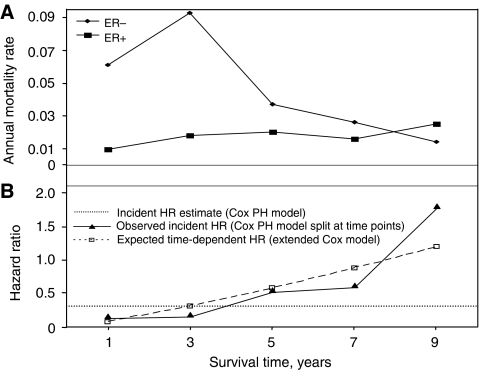
ER-specific annual mortality rates and hazard ratios by survival time. (**A**) Annual mortality rate for ER-negative and -positive tumours in incident cases by survival time in years. (**B**) Corresponding observed incident HRs for ER status (ER negative is referent) and the expected full cohort left-truncated HRs by survival time in years. The reference line shows the overall observed incident HR estimate calculated using a standard Cox proportional hazards model without adjustment for time-dependent effects. Observed incident HRs were calculated using a standard Cox proportional hazards model for incident cases only split at various time periods. Expected HRs were calculated using the extended Cox model formula at each year.

However, these differences might be expected if the PH assumption is violated; each prognostic factor had highly significant tests for PH assumption violation based on Schoenfeld residuals in model (c) (*P*<0.0001). The time-dependent effect of ER status is illustrated in Figure 2A, which shows the annual mortality rate for incident cases by ER status. Early after diagnosis, patients with ER-negative tumours experience higher mortality rates compared to ER-positive tumours; however, this difference becomes less with time and, after about 8 years after diagnosis, the annual mortality for women with ER-positive tumours is greater than for women with ER-negative tumours. The solid line in Figure 2B shows the corresponding HRs associated with ER status (ER-negative status is referent) estimated for different time periods based on the incident cases only, using a standard Cox PH model split at various time points. The HR is less than 1 before 8 years, showing the lower mortality rate associated with ER-positive tumours, but >1 after 8 years. Had the PH assumption held, the HR associated with ER status would be the same for various time points; this would be the overall estimated HR provided by the standard Cox model. However, in this case, the overall estimated HR for ER-positive tumours of 0.29 is effectively a weighted average of the different time-specific HRs (Figure 2B, dotted reference line). Thus the apparent difference between the parameter estimates under the different models is not the result of survival bias, but occurs because the PH assumption is violated. Under each model, the (time-independent) parameter estimate is a weighted average of the underlying time-specific parameter estimates, and as the number of subjects at risk at each time is different under the various models, the weighted averages will also be different.

### Analyses correcting for time-dependent effects

Cox models for stage, grade and ER status under the three scenarios were modified to include a covariate to allow for time-dependent effects (extended Cox model, Table 3). We found that the best-fit models allowed the *β*-coefficient to vary linearly with the natural logarithm of time (instead of varying linearly with time). Using the extended Cox formula, the HR at time *t* for time-varying covariates was calculated as 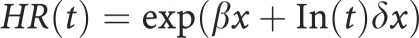 where *x* is the predictor variable, *β* is the *β*-coefficient and *δ* is the time-varying coefficient at time *t* (Kleinbaum and Klein, 2005). There were no significant differences between the *β*-coefficients for prognostic factors between the incident model and the models including prevalent cases. The test for interaction of case type and prognostic factor in model (c) was no longer significant for ER status (*P*=0.59) and remained non-significant for stage (*P*=0.06) and grade (*P*=0.07). The test for proportional assumption violation based on Schoenfeld residuals was no longer significant for any of the models.

The expected HRs for ER status at different times were derived from the parameter estimates from model (c) and are shown in Figure 2B (dashed line). These were very close to the observed time-specific HRs estimated using incident data only, suggesting that the extended Cox model fits the data well.

## Discussion

Studies with delayed entry – time between date of diagnosis (onset date) and study entry – are commonly encountered; these include observational epidemiological studies with prevalent cases. It is commonly thought that the use of prevalent cases in studies of disease prognosis results inevitably in bias. However, when the delay time is known, it can be factored into standard time-to-event analysis methods to provide valid tests of association between risk factor and event, as well as unbiased estimates of the HR associated with the risk factor. Our results, based on empirical data, demonstrate that unbiased estimates of the HR are obtained from a correctly specified model that adjusts for left truncation and time-dependent effects.

However, when using these methods for prognostic studies in prospective cohorts, it is important to note some cautions and limitations. If the current disease duration is not known (unknown date of diagnosis), the delayed entry estimator is not a viable option and biases in the parameter estimate can occur (Brookmeyer, 2005). If date of entry (age, time to blood draw) is used as a left truncation point, it does not need to be in the model as a covariate; in our example, prevalent case status would not need to be added to the left-truncated model (Klein and Moeschberger, 2003). Further, the methods that allow for left truncation rely on a key assumption: entry time and event time are conditionally independent, given the covariates included in the model. If this assumption is not met, the parameter estimates are not valid. Further discussion of the independent delayed entry assumption is provided by Keiding (1992).

Also, although left truncation methods allow the use of prevalent cases in survival analyses, the sample may represent a patient subset that is not generalisable to the patient population as a whole. In our example, we cannot extrapolate our results to individuals who died too quickly following diagnosis to be included in our study, either as prevalent or incident cases. Along these lines, care also needs to be taken in choosing an appropriate time scale. For example, when comparing treatment for a metastatic cancer, it would be inappropriate to measure survival from date of initial diagnosis, as we would not be able to make reasonable inferences on the disease population as a whole. A more appropriate onset date would be the date of diagnosis of advanced disease (Cnaan and Ryan, 1989).

A separate issue when working with a Cox PH model is possible PH assumption failure as when prognostic factors are time dependent; in other words, their hazards vary over time. The extended Cox PH model is adapted to deal with these types of covariates and can easily be performed with modern statistical packages. Our results show the importance of taking into account time-dependent effects in a Cox regression model when predictor variables violate the PH assumption in a study that includes prevalent and incident cases. In our example, all three prognosticators had statistically significant deviation from the PH assumption; this violation was no longer observed once we accounted for time-dependent effects. Also, besides inclusion of a time-varying coefficient, other valid methods for dealing with PH assumption failure exist, including stratification by the variable or multi-state modeling for Cox regression analysis, frailty models (Klein and Moeschberger, 2003) and misspecified Cox model analysis (Lin and Wei, 1989). It is important to note that interpretation of the HR can vary based on the technique used (Klein and Moeschberger, 2003).

In conclusion, observational epidemiological studies that include prevalent cases can provide a useful tool in the study of prognostic factors for disease, provided appropriate allowance for the prevalent ascertainment is made in the analysis. The major advantage of using prevalent cases is the gain in power from increased sample size which may be particularly important for the study of germ-line genetic predictors of outcome, where effect sizes are likely to be modest at best.

## Figures and Tables

**Table 1:** SEARCH participant survival and prognostic characteristics

	**Incident**	**Prevalent**	***P* a**
Total number subjects	1231	3239	
Total time at risk (years)	8517.1	16 532.2	
Median F/U (years)^b^	7.7 (0.48–10)^c^	7.2 (0.96–10)^c^	
Median time at risk (years)	7.3 (0.08–9.77)^c^	4.8 (0.03–9.48)^c^	
Median time from diagnosis to study entry (years)^d^	0.39 (0–0.5)^c^	1.84 (0.51–9.34)^c^	
Number of deaths	220	490	
Annual mortality rate	0.026	0.03	
5-year survival rate	0.88 (0.86–0.89)^e^	0.89 (0.88–0.91)^e^	
Median age at diagnosis, years	52 (25–65)^c^	51 (23–69)^c^	**0.001** f
			
*Age at diagnosis*			**<0.001** g
<40	89 (7.2%)	305 (9.4%)	
40–49	290 (23.6%)	1041 (32.1%)	
50–59	596 (48.4%)	1206 (37.2%)	
60+	256 (20.8%)	687 (21.2%)	
			
*Histopathological grade*			**0.25** g
Well differentiated	282 (22.9%)	592 (18.3%)	
Moderately differentiated	494 (40.1%)	1193 (36.8%)	
Poorly differentiated	320 (26.0%)	700 (21.6%)	
Unknown	135 (11.0%)	754 (23.3%)	
			
*Morphological type*			**0.006** g
Ductal	907 (73.7%)	2409 (74.4%)	
Lobular	206 (16.7%)	453 (14.0%)	
Other	103 (8.4%)	352 (10.9%)	
Unknown	15 (1.2%)	25 (0.8%)	
			
*Clinical stage*			**0.005** g
1	647 (56.6%)	1544 (47.7%)	
2	523 (42.5%)	1460 (45.1%)	
3 or 4	44 (3.6%)	150 (4.6%)	
Missing	17 (1.4%)	85 (2.6%)	
			
*ER*			0.41^g^
Negative	232 (18.9%)	417 (12.9%)	
Positive	671 (54.5%)	1304 (40.3%)	
Missing	328 (26.7%)	1518 (46.9%)	

ER=oestrogen receptor.

a Comparing incident and prevalent cases.

b Follow-up censored at 10 years.

c Range of variable.

d Allowing for left truncation.

e 95% CI.

f Two-tailed *t*-test.

g *χ*^2^-test. Bold values indicate a statistically significant test with *P*<0.05.

**Table 2:** Cox models for prevalent and incident breast cancer cases

**Prognostic factor**	**Model^a^**	***β-*Coefficient**	**(95% CL)^b^**	**Heterogeneity test *P*-value^c^**
Stage	Model (a)	1.15	(0.91, 1.38)	Ref
	Model (b)	0.95	(0.81, 1.10)	0.15
	Model (c)	1.01	(0.88, 1.13)	0.29
			*Case status interaction* P*-value*=*0.14*	
				
Grade	Model (a)	0.90	(0.68, 1.12)	Ref
	Model (b)	0.65	(0.50, 0.80)	0.07
	Model (c)	0.74	(0.61, 0.86)	0.21
			*Case status interaction* P*-value*=*0.07*	
				
ER Status	Model (a)	−1.25	(−1.56, −0.94)	Ref
	Model (b)	−0.52	(−0.80, −0.24)	**0.0006**
	Model (c)	−0.83	(−1.04, −0.63)	**0.03**
			*Case status interaction* P*-value*=***0.001***	

ER=oestrogen receptor.

a Each prognostic factor was modeled separately in a ‘univariate’ Cox model. Unknown or missing data for each prognostic variable were not included. Model (a): baseline model using incident cases only without allowing for left truncation; model (b): prevalent cases allowing for left truncation; model (c): all cases allowing for left truncation. A test for interaction of case status and prognostic factor was performed in model (c).

b Based on calculation of robust variances.

c Heterogeneity test comparing prognostic factor *β*-coefficient in models (b) and (c) to baseline model (a). Comparisons of *β*-coefficients from models (a) and (c) are not strictly valid as the models are not independent, but, where statistically significant, it demonstrates that estimates differ by more than 2 standard errors. Bold values indicate a statistically significant test with *P*<0.05.

**Table 3:** Time-dependent (extended) Cox models for prevalent and incident breast cancer cases

**Prognostic factor**	**Model^a^**	***β-*Coefficient**	**(95% CL)**	**Log-linear time-varying coefficient *δ***	**(95% CL)^b^**	**Heterogeneity test *P*-value^c^**
Stage	Model (a)	1.74	(1.22, 2.26)	−0.51	(−0.89, −0.13)	Ref
	Model (b)	1.95	(1.51, 2.39)	−0.61	(−0.87, −0.36)	0.55
	Model (c)	1.78	(1.45, 2.10)	−0.52	(−0.72, −0.32)	0.90
				*Case status interaction* P*-value*=*0.06*		
						
Grade	Model (a)	2.43	(1.61, 3.24)	−1.15	(−1.68, −0.63)	Ref
	Model (b)	1.70	(1.18, 2.22)	−0.66	(−0.96, −0.35)	0.14
	Model (c)	1.97	(1.55, 2.38)	−0.82	(−1.07, −0.57)	0.32
				*Case status interaction* P*-value*=*0.07*		
						
ER Status	Model (a)	−2.39	(−3.21, −1.56)	0.97	(0.36, 1.58)	Ref
	Model (b)	−2.38	(−3.24, −1.51)	1.27	(0.71, 1.84)	0.99
	Model (c)	−2.42	(−3.02, −1.82)	1.19	(0.78, 1.60)	0.95
				*Case status interaction* P*-value* = *0.59*		

ER=oestrogen receptor.

a Each prognostic factor was modeled separately in a ‘univariate’ Cox model. Unknown or missing data for each prognostic variable were not included. Model (a): baseline model using incident cases only without allowing for left truncation; model (b): prevalent cases allowing for left truncation; model (c): all cases allowing for left truncation. A test for interaction of case status and prognostic factor was performed in model (c).

b Based on calculation of robust variances.

c Heterogeneity test comparing prognostic factor *β-*coefficient only in models (b) and (c) to baseline model (a). Comparisons of *β-*coefficients from models (a) and (c) are not strictly valid as the models are not independent, but, where statistically significant, it demonstrates that estimates differ by more than 2 standard errors.

## References

[bib1] Azzato EM, Driver KE, Lesueur F, Shah M, Greenberg D, Easton DF, Teschendorff AE, Caldas C, Caporaso NE, Pharoah PD (2008) Effects of common germline genetic variation in cell cycle control genes on breast cancer survival: results from a population-based cohort. Breast Cancer Res 10: R47 18507837 10.1186/bcr2100 PMC2481496

[bib2] Barnett GC, Shah M, Redman K, Easton DF, Ponder BA, Pharoah PD (2008) Risk factors for the incidence of breast cancer: do they affect survival from the disease? J Clin Oncol 26: 3310–3316 18612147 10.1200/JCO.2006.10.3168

[bib3] Brookmeyer R (2005) Biased sampling of cohorts. In Encyclopedia of Biostatistics, Armitage P, Colton T (eds) John Wiley: Hoboken, NJ

[bib4] Cnaan A, Ryan L (1989) Survival analysis in natural history studies of disease. Stat Med 8: 1255–1268 2814073 10.1002/sim.4780081009

[bib5] Goode EL, Dunning AM, Kuschel B, Healey CS, Day NE, Ponder BA, Easton DF, Pharoah PP (2002) Effect of germ-line genetic variation on breast cancer survival in a population-based study. Cancer Res 62: 3052–3057 12036913

[bib6] Grambsch PM, Therneau TM (1994) Proportional hazards tests and diagnostics based on weighted residuals. Biometrika 81: 515–526

[bib7] Harvey JM, Clark GM, Osborne CK, Allred DC (1999) Estrogen receptor status by immunohistochemistry is superior to the ligand-binding assay for predicting response to adjuvant endocrine therapy in breast cancer. J Clin Oncol 17: 1474–1481 10334533 10.1200/JCO.1999.17.5.1474

[bib8] Keiding N (1992) Independent delayed entry. In Survival Analysis: State of the Art, Klein JP, Goel PK (eds), pp 309–326. Kluwer: Dordrecht

[bib9] Keiding N (2005) Delayed entry. In Encyclopedia of Biostatistics, Armitage P, Colton T (eds) John Wiley: Hoboken, NJ

[bib10] Klein JP, Moeschberger ML (2003) Survival Analysis: Techniques for Censored and Truncated Data, 2nd edn Springer: New York

[bib11] Kleinbaum DG, Klein M (2005) Survival Analysis: A Self-learning Text, 2nd edn Springer: New York

[bib12] Lin DY, Wei LJ (1989) The robust inference for the Cox proportional hazards model. JASA 84: 1074–1078

[bib13] Sobin LH, Wittekind C (1997) TNM Classification of Malignant Tumours, 5th edn Wiley-Liss: New York

[bib14] Song H, Hogdall E, Ramus SJ, Dicioccio RA, Hogdall C, Quaye L, McGuire V, Whittemore AS, Shah M, Greenberg D, Easton DF, Kjaer SK, Pharoah PD, Gayther SA (2008) Effects of common germ-line genetic variation in cell cycle genes on ovarian cancer survival. Clin Cancer Res 14: 1090–1095 18281541 10.1158/1078-0432.CCR-07-1195 PMC2430032

[bib15] Udler M, Maia AT, Cebrian A, Brown C, Greenberg D, Shah M, Caldas C, Dunning A, Easton D, Ponder B, Pharoah P (2007) Common germline genetic variation in antioxidant defense genes and survival after diagnosis of breast cancer. J Clin Oncol 25: 3015–3023 17634480 10.1200/JCO.2006.10.0099

